# Supplementary material for: The First High-Quality Genome Assembly of Freshwater Pearl Mussel Sinohyriopsis cumingii: New Insights into Pearl Biomineralization
Source: Int J Mol Sci. 2024 Mar 9;25(6):3146. doi: 10.3390/ijms25063146 (PMC10969987; doi:10.3390/ijms25063146)
Supplement: Supplementary file 1 [file ijms-25-03146-s001.zip › Supplemental figures.pdf]

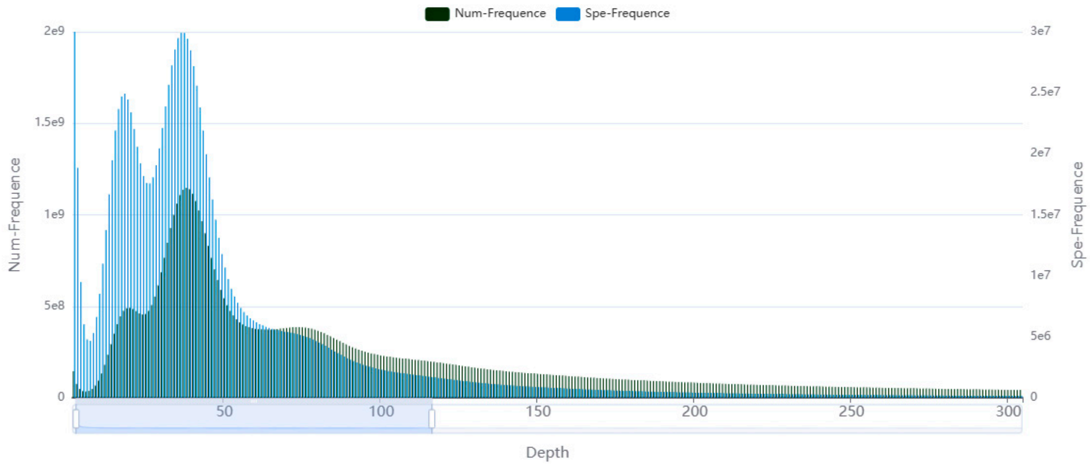

Figure S1. 17-mer distribution chart.

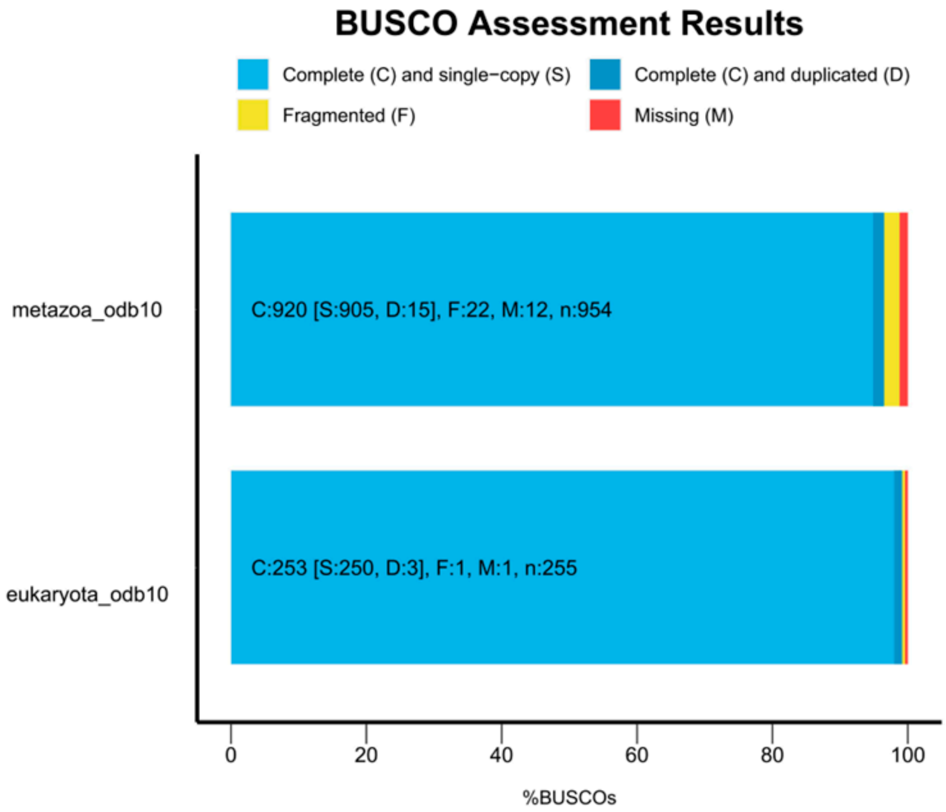

Figure S2. Core gene estimation for *S. cumingii* assembly using BUSCO.

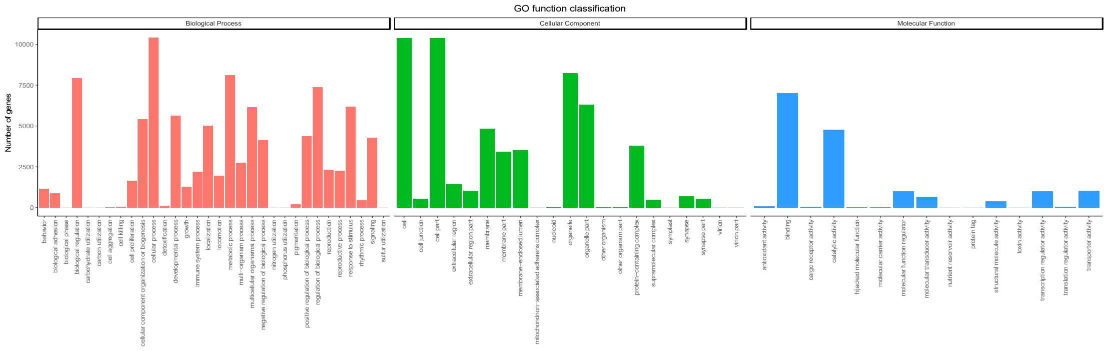

Figure S3. GO analysis and functional classification of the protein coding genes in *S. cumingii*.

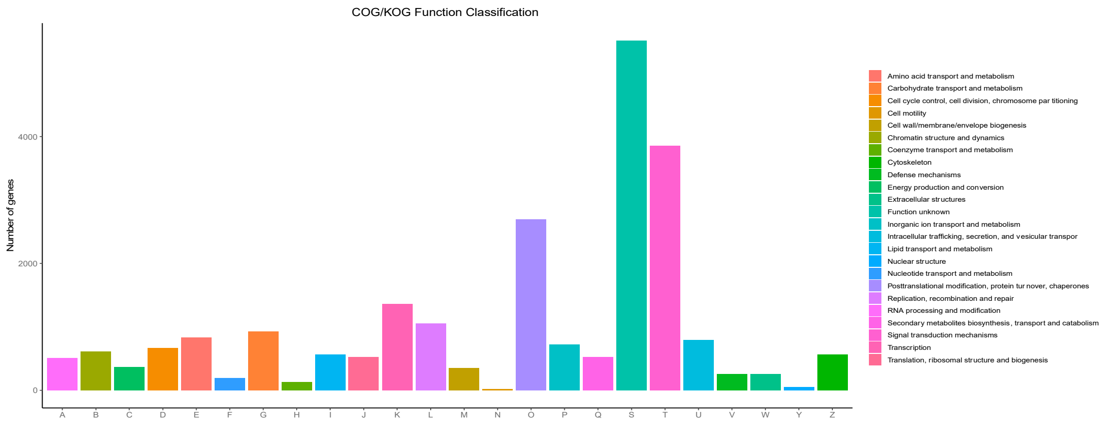

Figure S4. COG analysis and functional classification of the protein coding genes in *S. cumingii*.

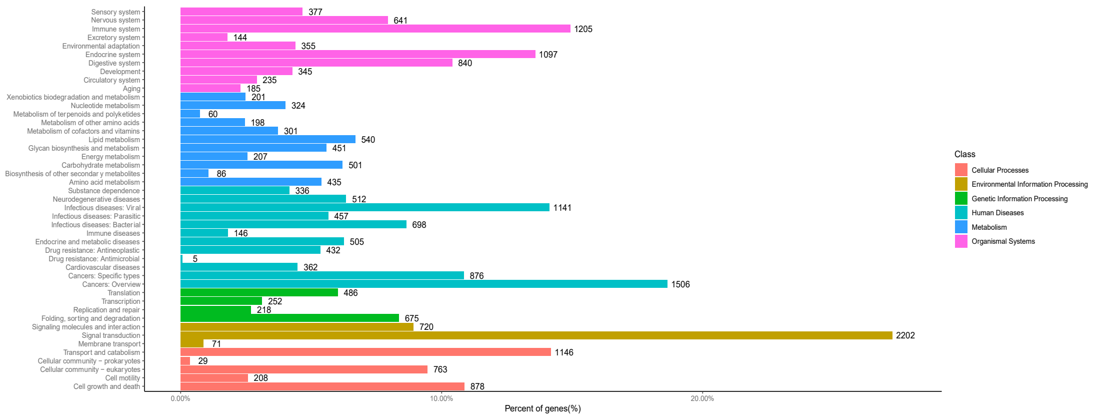

Figure S5. KEGG analysis and functional classification of the protein coding genes in *S. cumingii*.

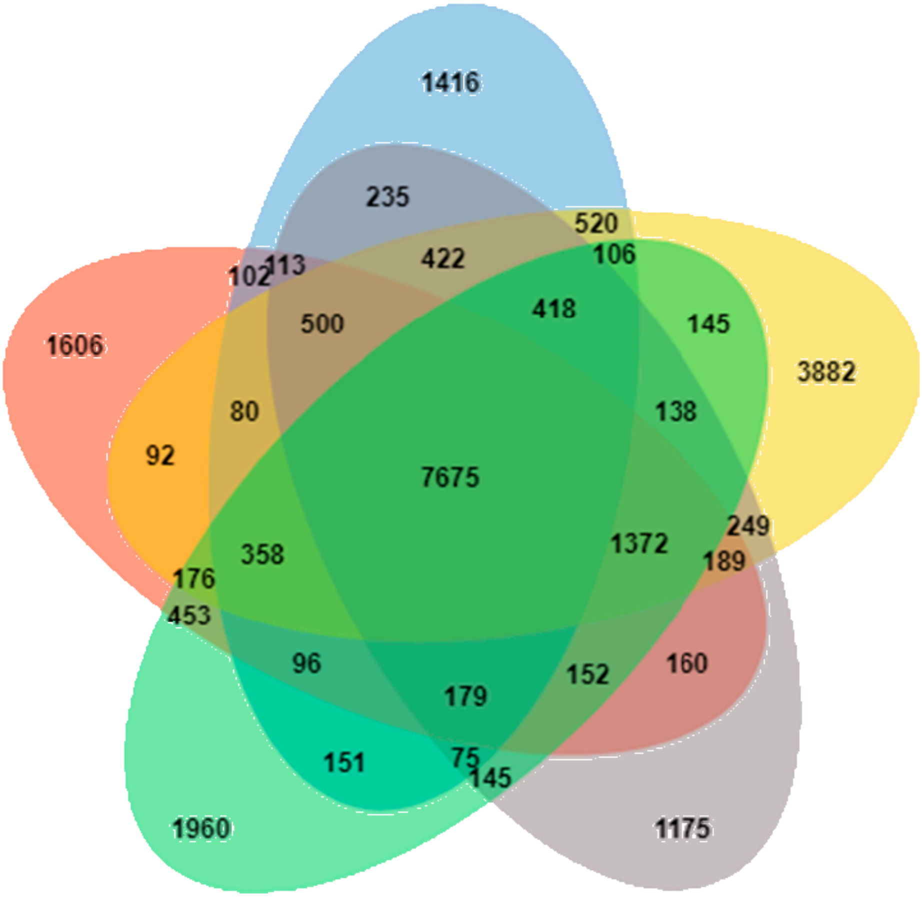

Figure S6. Upsetplot of geneorthologues shared among *S. cumingii, P. fucata, C. gigas, C. farreri* and *D. rostriformis*.

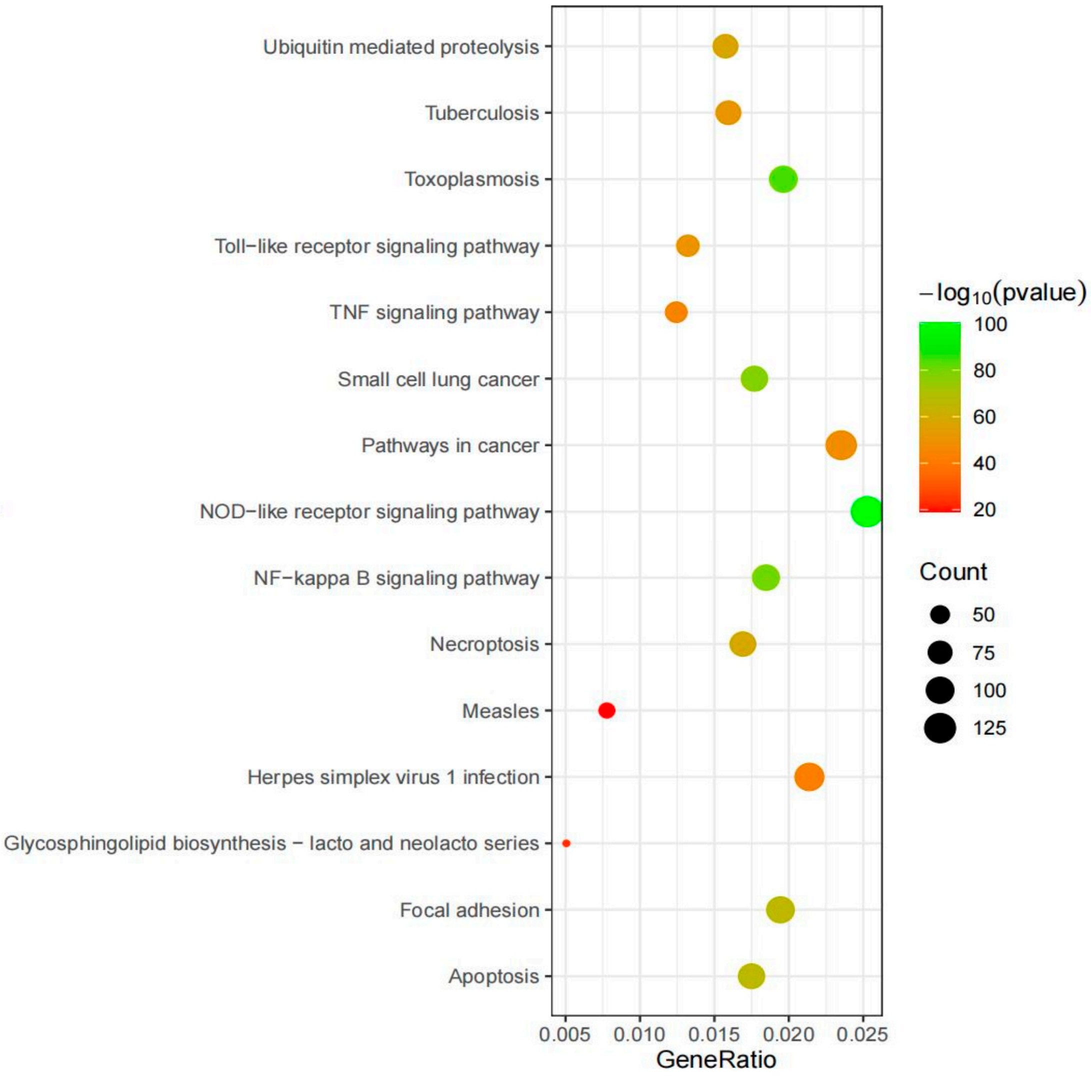

Figure S7. Top fifteen KEGG pathway enrichment of *S. cumingii*-specific gene families.

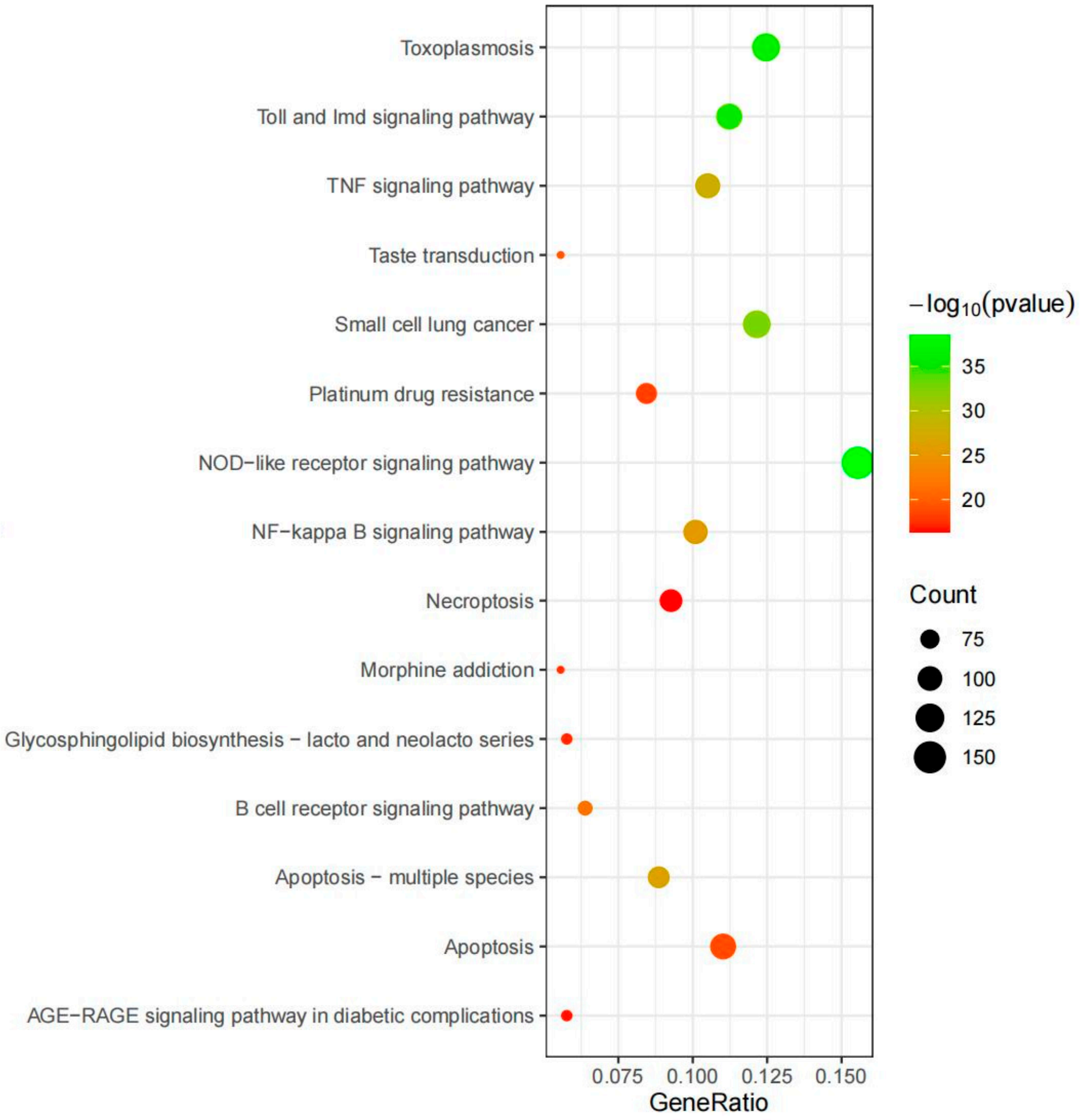

Figure S8. Top fifteen KEGG pathway enrichment of expanded gene families in *S. cumingii* genome.

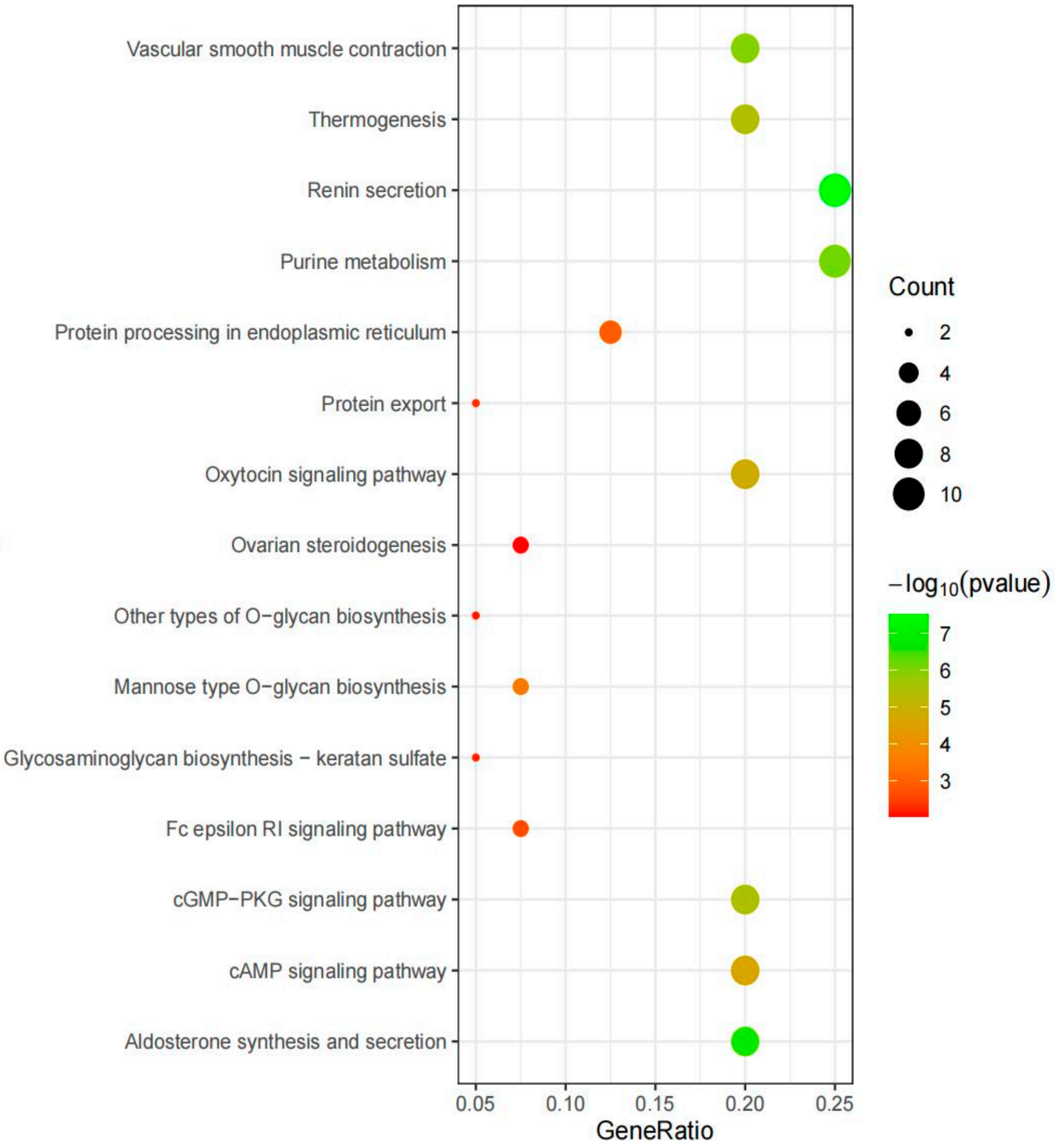

Figure S9. Top fifteen KEGG pathway enrichment of constricted gene families in *S. cumingii* genome.

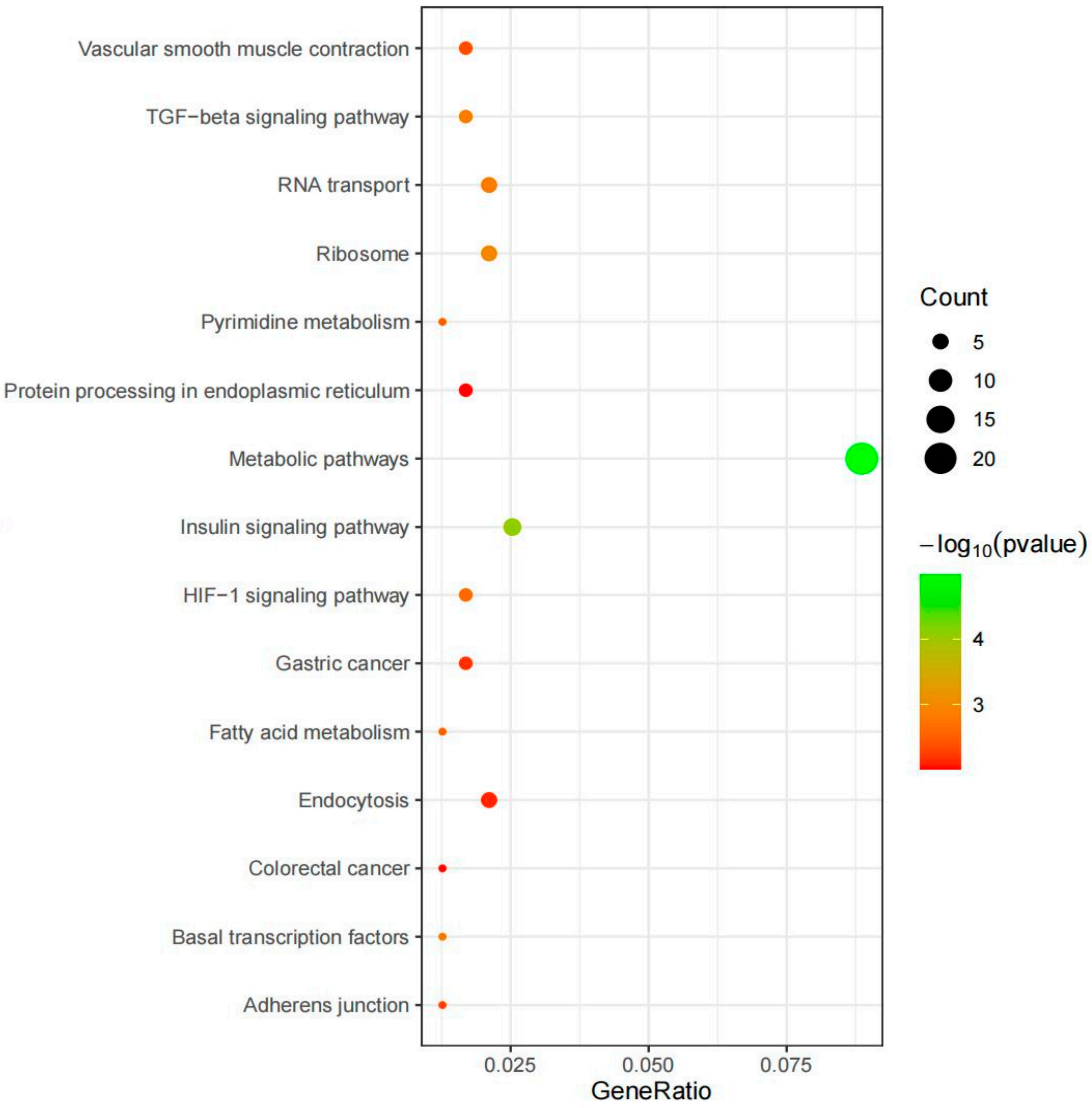

Figure S10. Top fifteen KEGG pathway enrichment of positive selection genes in *S. cumingii* genome.

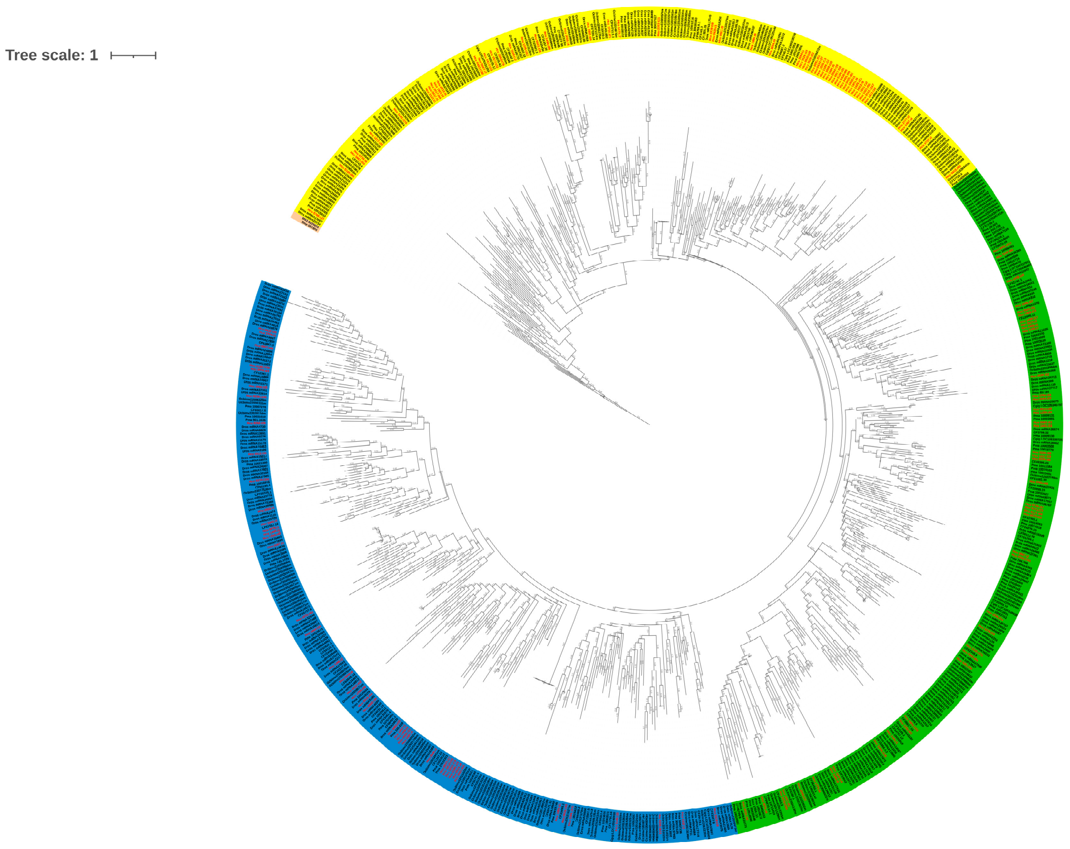

Figure S11. Maximum likelihood phylogenetic tree of fibrillins. Different color strip represents different clusters. Red-bond labels represent gene ID of *S. cumingii*.

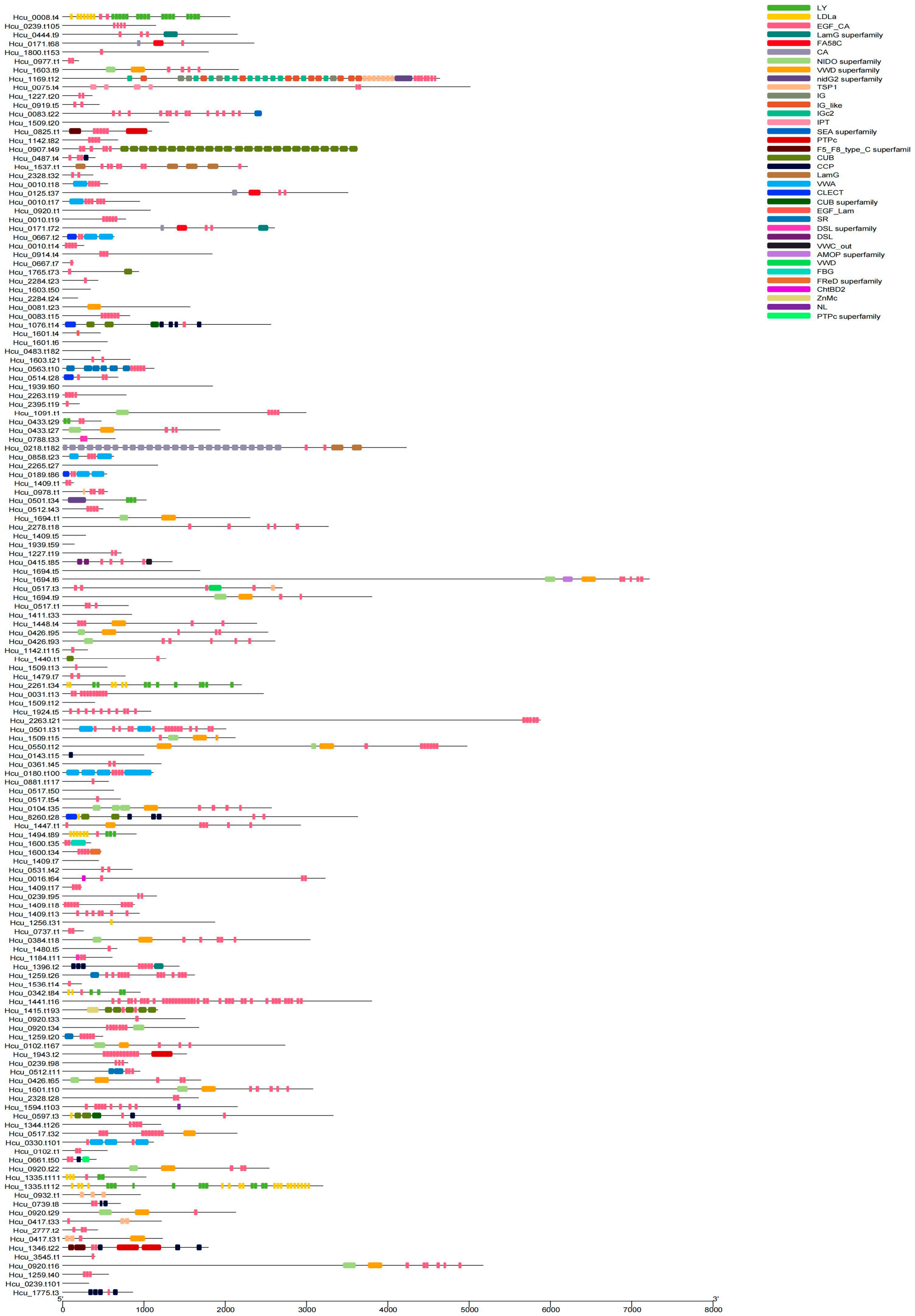

Figure S12. Conversed domains of fibrillins in *S. cumingii* genome.

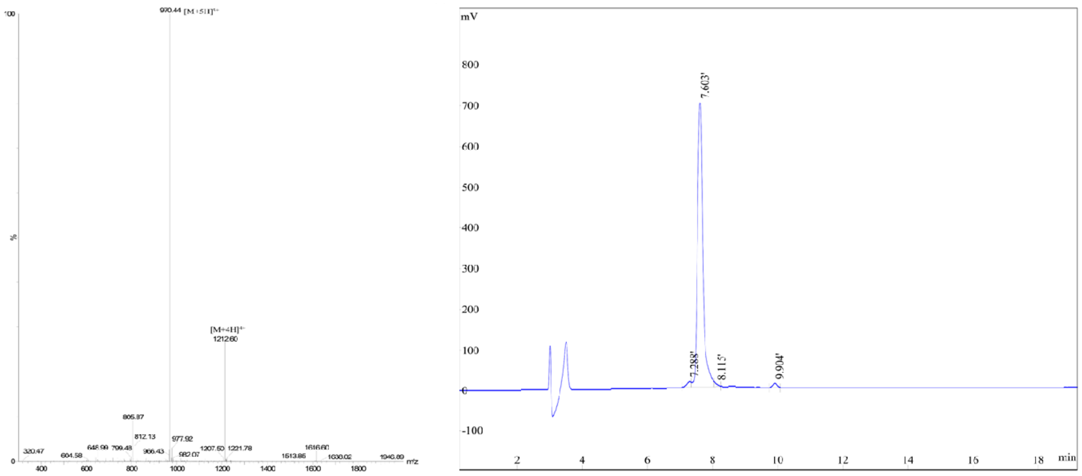

Figure S13. MS and HPLC analysis of peptide with conserved EGF-CA domain.
